# Brain tumor segmentation using deep learning: high performance with minimized MRI data

**DOI:** 10.3389/fradi.2025.1616293

**Published:** 2025-07-08

**Authors:** Jacky Huang, Banu Yagmurlu, Powell Molleti, Richard Lee, Abigail VanderPloeg, Humaira Noor, Rohan Bareja, Yiheng Li, Michael Iv, Haruka Itakura

**Affiliations:** ^1^ Department of Medicine, Division of Oncology, Stanford University School of Medicine, Stanford, CA, United States; ^2^ Department of Radiology, Stanford University School of Medicine, Stanford, CA, United States; ^3^ Department of Medicine, Stanford Center for Biomedical Informatics Research, Stanford University School of Medicine, Stanford, CA, United States

**Keywords:** artificial intelligence, deep learning (DL), computer vision, convolutional neural network (CNN), 3D brain tumor segmentation, semantic segmentation, glioma, magnetic resonance imaging (MRI)

## Abstract

**Purpose:**

Brain tumor segmentation with MRI is a challenging task, traditionally relying on manual delineation of regions-of-interest across multiple imaging sequences. However, this data-intensive approach is time-consuming. We aimed to optimize the process by using a deep learning (DL) based model while minimizing the number of MRI sequences required to segment gliomas.

**Methods:**

We trained a 3D U-Net DL model using the annotated 2018 MICCAI BraTS dataset (training dataset, *n* = 285), focusing on sub-segmenting enhancing tumor (ET) and tumor core (TC). We compared the performances of models trained on four different combinations of MRI sequences: T1C-only, FLAIR-only, T1C + FLAIR and T1 + T2 + T1C + FLAIR to evaluate whether a smaller MRI data subset could achieve comparable performance. We evaluated the performance on the four different sequence combinations using 5-fold cross-validation on the training dataset, then on our test dataset (*n* = 358) consisting of samples from a separately held-out 2018 BraTS validation set (*n* = 66) and 2021 BraTS datasets (*n* = 292). Dice scores on both cross-validation and test datasets were assessed to measure model performance.

**Results:**

Dice scores on cross-validation showed that T1C + FLAIR (ET: 0.814, TC: 0.856) matched or outperformed those of T1 + T2 + T1C + FLAIR (ET: 0.785, TC: 0.841), T1C-only (ET: 0.781, TC: 0.852) and FLAIR-only (ET: 0.008, TC: 0.619). Results on the test dataset also showed that T1C + FLAIR (ET: 0.867, TC: 0.926) matched or outperformed those of T1 + T2 + T1C + FLAIR (ET: 0.835, TC: 0.908), T1C-only (ET: 0.726, TC: 0.928), and FLAIR-only (ET: 0.056, TC: 0.543). T1C + FLAIR excelled in both ET and TC, exceeding the performance of the four-sequence dataset. T1C-only matched T1C + FLAIR in TC performance. Similarly**,** T1C and T1C + FLAIR also outperformed in ET delineation by sensitivity (0.829) and Hausdorff distance (5.964) on the test set. Across all configurations, specificity remained high (≥0.958). T1C performed well in TC delineation (sensitivity: 0.737), but the inclusion of all sequences led to improvement (0.754). Hausdorff distances clustered in a narrow range (17.622–33.812) for TC delineation across the configurations.

**Conclusions:**

DL-based brain tumor segmentation can achieve high accuracy using only two MRI sequences (T1C + FLAIR). Reduction of multiple sequence dependency may enhance DL generalizability and dissemination in both clinical and research contexts. Our findings may ultimately help mitigate human labor intensity of a complex task integral to medical imaging analysis.

## 1 Introduction

In managing brain tumors, differentiating viable tumor from necrosis or peritumoral edema and accurately delineating tumor margins are crucial for staging, treatment planning, monitoring tumor growth, assessing treatment response, and informing clinical outcomes, such as survival. Accurate tumor segmentation discriminates the pathologic lesion from the surrounding tissue, and extraction of tumor-specific features from the segmentation have had utility in correlating with tumor biomarkers and predicting clinical outcomes (1). However, manual tumor segmentation is time-intensive and subject to inter-operator variability (2, 3). To facilitate the implementation of segmentation tasks in the clinical setting, artificial intelligence (AI)-based computational models, such as deep learning (DL) algorithms are increasingly being applied in research settings (3–7). Developing accurate tumor segmentation algorithms is a complex problem due to substantial spatial and structural variability among brain tumors, along with the challenges of segmenting diffusely infiltrating tumors, such as gliomas (8). In addition, building an algorithm that can successfully segment both high- and low-grade gliomas (HGGs and LGGs) is challenging (8, 9). Furthermore, the requirement for large datasets in training DL algorithms can be burdensome.

The requirement for large datasets is typified in the Brain Tumor Segmentation (BraTS) Challenges (10) which represent some of the highest standards for evaluating and benchmarking evolving DL methods for brain tumor segmentation tasks. To examine different methodologic approaches, the combination of four brain magnetic resonance imaging (MRI) sequences (T1 + T1C + T2 + FLAIR) are commonly used as an accepted requisite for achieving high performances (11–13) in DL models. However, the burden of the full-sequence dataset creates a barrier to technology dissemination and practical applicability in the real-world. We thus hypothesized that a reduced sequence dataset could achieve comparable performance as the full dataset and sought to find the most informative minimal subset of MRI sequences.

First, rather than exhaustively evaluate all possible combinations, particularly those that were unlikely to yield clinically meaningful results (e.g., non-enhanced T1 and T2 without FLAIR), we chose to prioritize T1C and FLAIR as these sequences have individually demonstrated high tumor delineation capabilities both in clinical practice (13) and in our own work. Second, we fixed the methodological algorithm and varied only the MRI configurations. Convolutional Neural Networks (CNNs) have represented the state-of-the-art technology for brain tumor segmentation tasks, and U-Net, a CNN, has long been a standard in computer vision, excelling in image classification and segmentation tasks. Indeed, U-Net based architecture, along with its variants, have dominated as perennial winners of the BraTS Challenge over the years (14). Vision Transformers, which are based on the Transformer architecture originally designed for natural language processing, have become a powerful alternative for such computer vision tasks (15). However, their disadvantageous features make them less suitable for our study, including the requirement for large datasets to perform well, a tendency to overfit on smaller datasets, and the higher computational cost compared with the U-Net. In contrast, U-Net was considered well-suited for our study objective given its high performance on smaller datasets and computational efficiency – both features that promote usability in resource-constrained real-world environments (16).

We chose the 3D U-Net (17), a commonly used DL architecture and a variation of the Fully Convolutional Network (18), designed for biomedical image segmentation (19, 20). Using this architecture, we performed semantic segmentation by labeling every voxel of tumor region as tumor core (TC) or enhancing tumor (ET). TC comprised both enhancing and non-enhancing tumor subregions.

Third, we leveraged the high-quality, annotated brain tumor segmented datasets provided by BraTS, representing a mixed population of HGGs and LGGs**.** For training and validating the 3D U-Net algorithm, we used the 2018 and 2021 datasets from BraTS (10, 21), which are benchmarked as high-quality, standardized, neuroradiologist-annotated brain tumor data approved by the MICCAI Society (4, 22). Each dataset consisted of four brain MRI sequences (T1, T1C, T2, FLAIR). We tested four different experimental conditions, or subsets of sequences, to determine the minimum number of sequence data necessary for training the 3D U-Net that can achieve high accurate segmentation on our test dataset. We compared the final performances of the 3D U-Net (23) trained on the four different subsets of sequences by reporting their respective performances on the test dataset. For the 2018 dataset, we the built-in evaluation portal used by BraTS was available for blinded provision of performance metrics of our completed tumor segmentations. To our knowledge, only one other prior work using BraTS dataset was identified that also endeavored to identify the minimum dataset for achieving accurate tumor segmentation (13).

In this retrospective study, our aim was to identify the minimum number of MRI sequences needed for training a DL algorithm capable of achieving acceptably accurate segmentations in gliomas. The overarching objective was to enhance practical applicability, generalizability, and dissemination of an impactful technology in the real-world setting.

## 2 Materials and methods

### 2.1 Dataset

**Figure 1:**
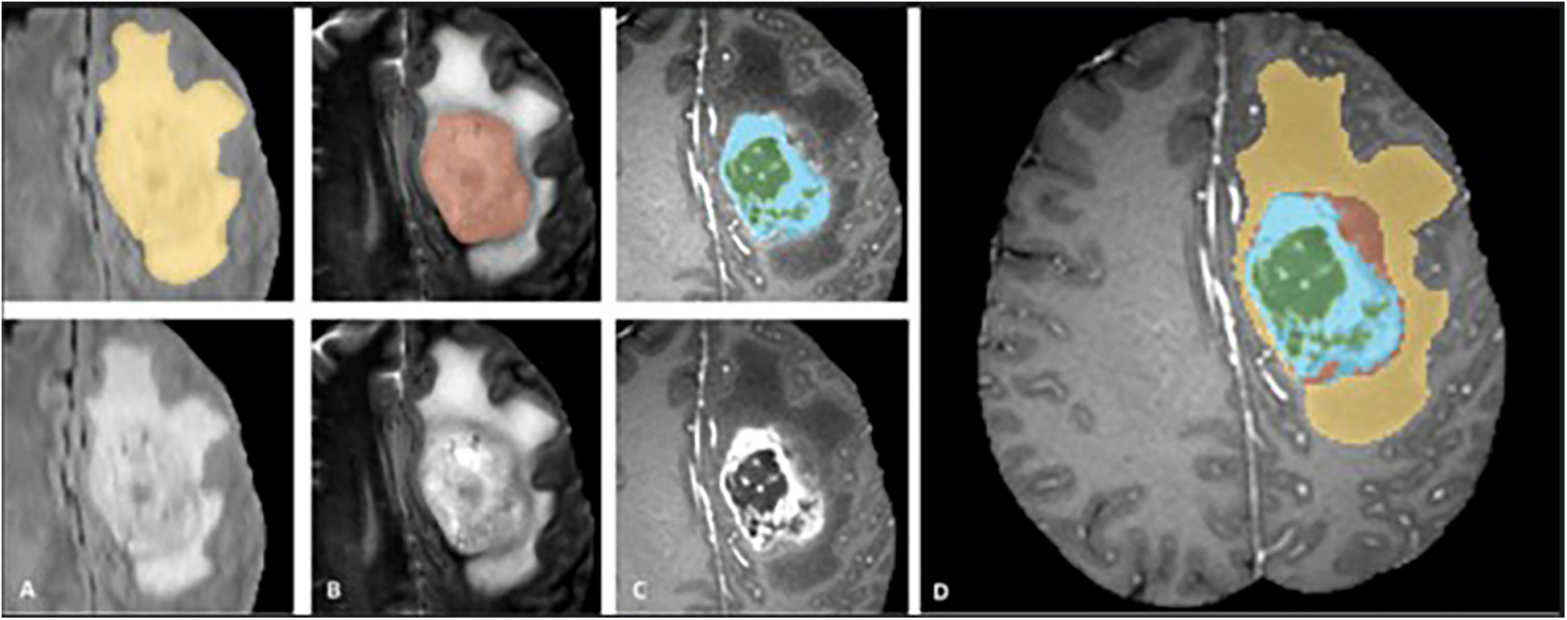
Manual ground truth annotations in the braTS training dataset showing tumor subregions in image patches (top left) (FLAIR, T2, T1C from **(A–C)**, respectively) and native images with corresponding sequences bottom left, **(A–C)**. Image patches from left to right; whole tumor consisting of TC and edema together (yellow) in FLAIR, TC (solid and cystic-necrotic core together) (red) in T2, enhancing tumor (ET) (blue) and necrotic-cystic core (green) in T1C. Final labels for the whole dataset combined (right) **(D)** shows ET (blue), cystic-necrotic core (green), non-enhancing solid core (red) and edema (yellow). © 20XX IEEE. Reprinted with permission from IEEE Transactions on Medical Imaging from “The Multimodal Brain Tumor Image Segmentation Benchmark (BRATS)” by Menze et al. (4).

We used multi-sequence MRI data from the MICCAI BraTS 2018 and BraTS 2021 datasets with four sequences available (T1, T2, FLAIR, T1C). We excluded cases that had missing sequences in the BraTS 2021 dataset. Our training dataset consisted of 285 glioma cases (210 HGGs and 75 LGGs) from the BraTS 2018 dataset (10). The test dataset consisted of 358 patients, including 66 cases from the BraTS 2018 validation dataset, combined with 292 cases from BraTS 2021. Unlike BraTS 2018, grades and molecular markers of the tumor were not determined in BraTS 2021 (21). Except for the 66 cases from BraTS 2018, all data were accompanied by ground-truth segmentations provided by MICCAI, delineating semantic classifications of tumor core (TC), enhancing tumor (ET), cystic-necrotic core, non-enhancing solid tumor core, and edema as shown in Figure 1 (4). Our study focused on segmenting TC and ET, which were subregions that could be evaluated by the portal, and treated them as independent problems by training a separate set of models for each.

### 2.2 Data processing

BraTS imaging data were partially preprocessed (3) and skull-stripped to remove non-brain parenchymal structures for enhanced training efficiency. The resolution of the scans was interpolated to 1mm^3^ per voxel. Since the individual patient MRI studies were acquired from different institutions, scanners, and protocols, we Z-normalized each image to have zero mean and unit variance. We employed commonly applied data augmentation techniques, including rotations, translations, image flipping and intensity scaling. Finally, we applied one-hot encoding to the ground truth.

### 2.3 Tumor segmentation algorithm

We trained models based on the 3D U-Net (17) which is an encoder-decoder style architecture with contraction layers that capture latent information about the MRI scan, followed by expansion layers which create an output mask. We chose not to make major modifications to the baseline U-Net architecture (17) and utilized the standard depth of contraction and expansion layers of 4. Initialization of model parameters was done through randomization. Filter size was initially set to 32, doubled at each contraction layer up to 16×, and halved at each expansion layer. The kernel size and the stride were set to 3 × 3 × 3 and 2 × 2 × 2, respectively, and we added BatchNorm and Dropout (with 0.5 probability) after each convolutional layer to combat overfitting. A final 1 × 1 × 1 convolution with softmax activation and filter size 2 produced a probability distribution for each voxel in the scan representing the probability of that voxel being tumor. The predicted segmentation mask was then obtained by taking the argmax of these probabilities.

**Figure 2:**
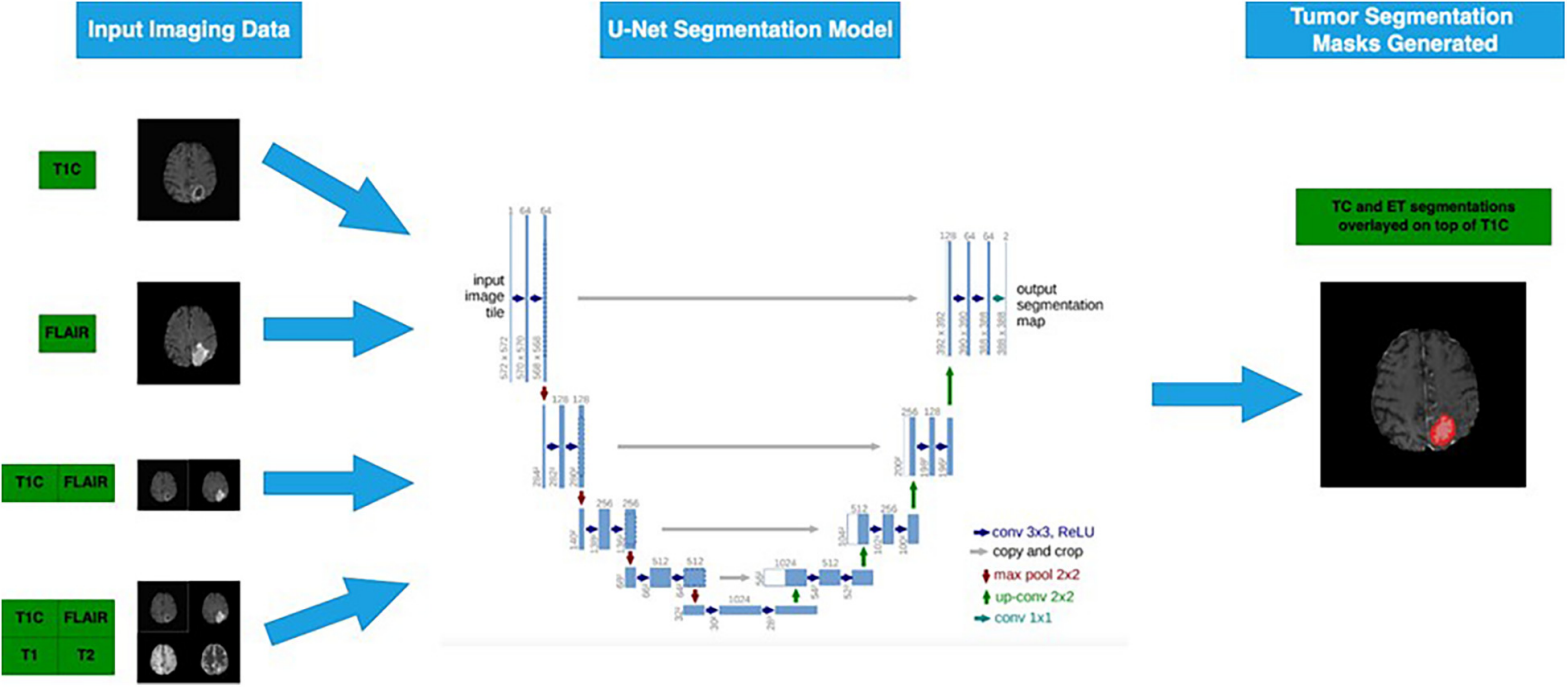
Four different subsets of the four MRI sequences are passed into the 3D U-Net model to generate segmentation masks for ET and TC sub-regions (6). Flowchart illustrating a U-Net segmentation model for tumor detection. Input imaging data includes T1C and FLAIR MRI scans.

We treated each individual voxel as a binary classification problem (e.g., ET or not, TC or not) and encoded any tumor-containing voxel representing the subregion of interest into the same value. Aligning our segmentation tasks to ET and TC regions allowed Dice score evaluation on the test dataset using our ground truth labels on or the CBICA portal for the 66 cases. For each tumor segmentation task, we trained on four different experimental configurations, representing four different sets of input MRI sequences (Figure 2) (17).

### 2.4 Model training

We conducted patch-based training on our 3D U-Net model with sampled sub-volumes (patches) of samples on the training dataset. Each patch had dimensions 80 × 80 × 80, and one epoch evaluated 3,000 patches. Our patch selection algorithm consisted of randomly selecting a single 3D image with replacement from the training set, followed by randomly sampling with replacement an 80 × 80 × 80 patch from the scan. To guarantee that the model was not learning from patches that contained only background voxels, we discarded patches with less than 100 tumor-containing voxels. We trained with a batch size of 2 patches. Patch and batch sizes were selected through trial and error to optimize two parameters: ability to fit within a single NVIDIA GeForce RTX 2080 Ti GPU while achieving sizes that enabled efficient learning and generalizability with adequate spatial context. Other hyperparameter-tuning utilized cross-validation. Adam optimizer with 0.0001 learning rate was used, along with the Dice coefficient loss function. We trained to 60 epochs, generating checkpoints after every 5 epochs for evaluation.

### 2.5 Statistical analysis

We performed 5-fold cross-validation and calculated the median Dice score across all the samples in each held-out fold at epoch 60. Dice score, a generally accepted metric for segmentation tasks (24) that measures the overlap between two sets X and Y was calculated for each fold using the formula:

$$\mathrm{Dice}\;\mathrm{Score}=\frac{2|X\cap Y|}{\left|X|+|Y\right|}$$

where X and Y represent our segmentation mask and the ground truth, respectively. To evaluate the model performance on the training dataset, we computed the mean of the median Dice scores across the five folds in cross-validation. We compared performances across multiple groups using a one-way ANOVA, alpha = 0.05, assuming unequal variances, on the cross-validation results. We retrained each model with the full training dataset, then applied each on the test dataset (*n* = 358) to assess performance. Of the test dataset, 292 cases had accompanying ground truth segmentations by which segmentation performance accuracy was assessed and Dice scores calculated. The remaining 66 cases were unannotated, and Dice scores were assessed through the CBICA portal hosted by MICCAI (25). The portal compares uploaded segmentations with their blinded ground truths and returns performance metrics for each sample, including Dice scores. As a secondary set of comparisons, we also evaluated sensitivity, specificity, and 95% Hausdorff distance.

## 3 Results

**Figure 3:**
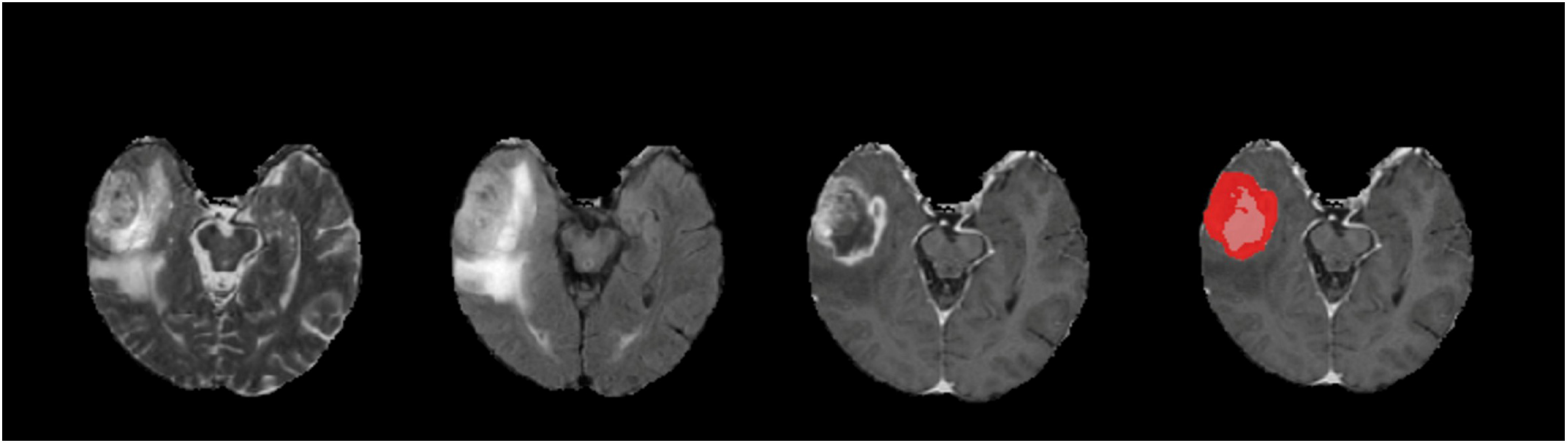
An example of a tumor segmentation on a right temporal lobe glioma from the braTS 2018 validation dataset. Demonstrated from left to right are native T2, FLAIR, T1C, and predicted tumor segmentation masks generated for ET (red) and TC (red and pink superimposed). For this case, our T1C + FLAIR models yielded Dice scores of 0.871 and 0.916 for ET and TC segmentations, respectively.

We visually examined generated tumor masks to evaluate our models qualitatively. Figure 3 is a rendition of generated tumor sub-segmentations of a single sample from the BraTS 2018 validation dataset (20), showing raw T2, FLAIR, T1C images of a glial tumor as well as the tumor segmentation mask with superimposition of ET and TC.

Dice scores resulting from cross-validation on the training dataset and from the test datasets on the four sequence subset combinations are summarized in Table 1. Notably, while segmentation using T1C-only performed well for TC, matching the performance of T1C + FLAIR, the best overall performance was obtained using the T1C + FLAIR combination. This configuration achieved the highest Dice score for ET segmentation and slightly improved the TC result compared to the full four-sequence input.

**Table 1:** Summary of ET (enhancing tumor) and TC (tumor core) segmentation performances by 5-fold cross-validation on the training data and on the test dataset.

Segmentation type	Sequence subset	Median dice score on the training dataset with 5-fold cross-validation (± 95% CI)	Median dice score on the test dataset
Enhancing tumor	T1C	0.781 (0.09)	0.726
Enhancing tumor	FLAIR	0.008 (0.01)	0.056
Enhancing tumor	Doublet	0.814 (0.01)	0.867
Enhancing tumor	Quadruplet	0.785 (0.04)	0.835
Tumor core	T1C	0.852 (0.03)	0.928
Tumor core	FLAIR	0.619 (0.07)	0.543
Tumor core	Doublet	0.856 (0.02)	0.926
Tumor core	Quadruplet	0.841 (0.03)	0.908

CI, Confidence Interval.

Results are median dice scores at epoch 60 using four experimental imaging configurations (T1C-only, FLAIR-only, T1C + FLAIR doublet, and T1 + T2 + T1C + FLAIR quadruplet).

**Figure 4:**
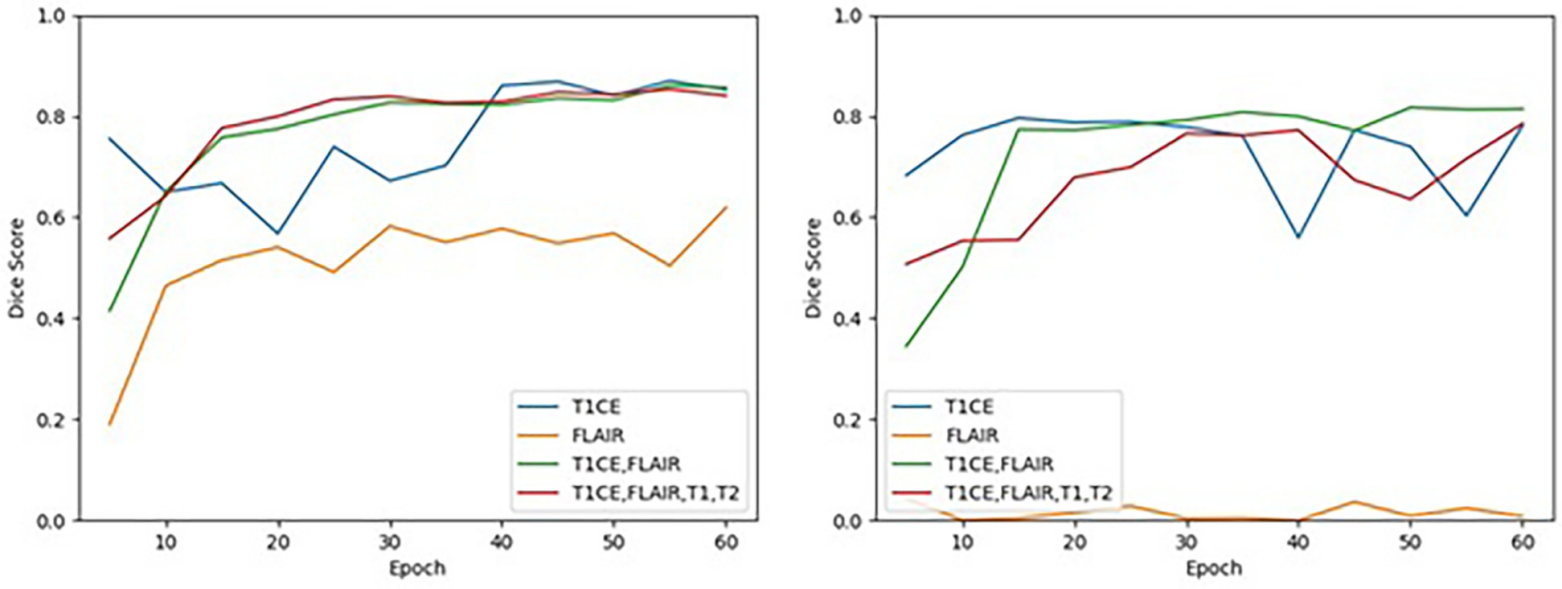
Cross-validation results on the training dataset for TC (left) and ET (right) segmentation for each MRI sequence combination by epoch. Performance of TC and ET segmentation, as measured by the mean of the median Dice scores across the 5 folds, is shown for each MRI sequence combination at an interval of every 5 epochs.

All models, except single sequence FLAIR, achieved good performances in both ET and TC segmentation. In ET segmentation (Table 1 and Figure 4, right panel), no significant difference was noted among single sequence T1C-only, T1C + FLAIR (doublet), and T1 + T2 + FLAIR + T1C (quadruplet) in both cross-validation on the training dataset (Dice scores of 0.781, 0.814, and 0.785, respectively; one-way ANOVA *p* = 0.33), and the test dataset (Dice scores 0.726, 0.867, and 0.835, respectively). In contrast, FLAIR-only achieved low Dice scores of 0.008 and 0.056, respectively. In TC segmentation (Table 1; Figure 4, left panel, T1C performance (Dice score 0.852) paralleled those of the T1C + FLAIR doublet (Dice score 0.856) and T1C + FLAIR + T1 + T2 quadruplet (Dice score 0.841) in cross-validation, as well as in the test dataset (Dice scores 0.928, 0.926, and 0.908, respectively). Single sequence FLAIR lagged in performance in both cross-validation of the training dataset (Dice score 0.619) and on the test dataset (Dice score 0.543), although it performed slightly better than in ET.

The T1C + FLAIR doublet was able to perform to the same level or even higher as any other combination of sequences on both the ET and the TC at epoch 60. Test data results paralleled those of cross-validation training data results across all models. Among the test data results, T1C + FLAIR doublet was a consistently top performer compared to other models in both ET and TC segmentation (Table 1). The computational time required per epoch for a single, doublet, and quadruplet set of sequences was 7.5 minutes, 9.5 minutes, and 11 minutes, respectively.

**Figure 5:**
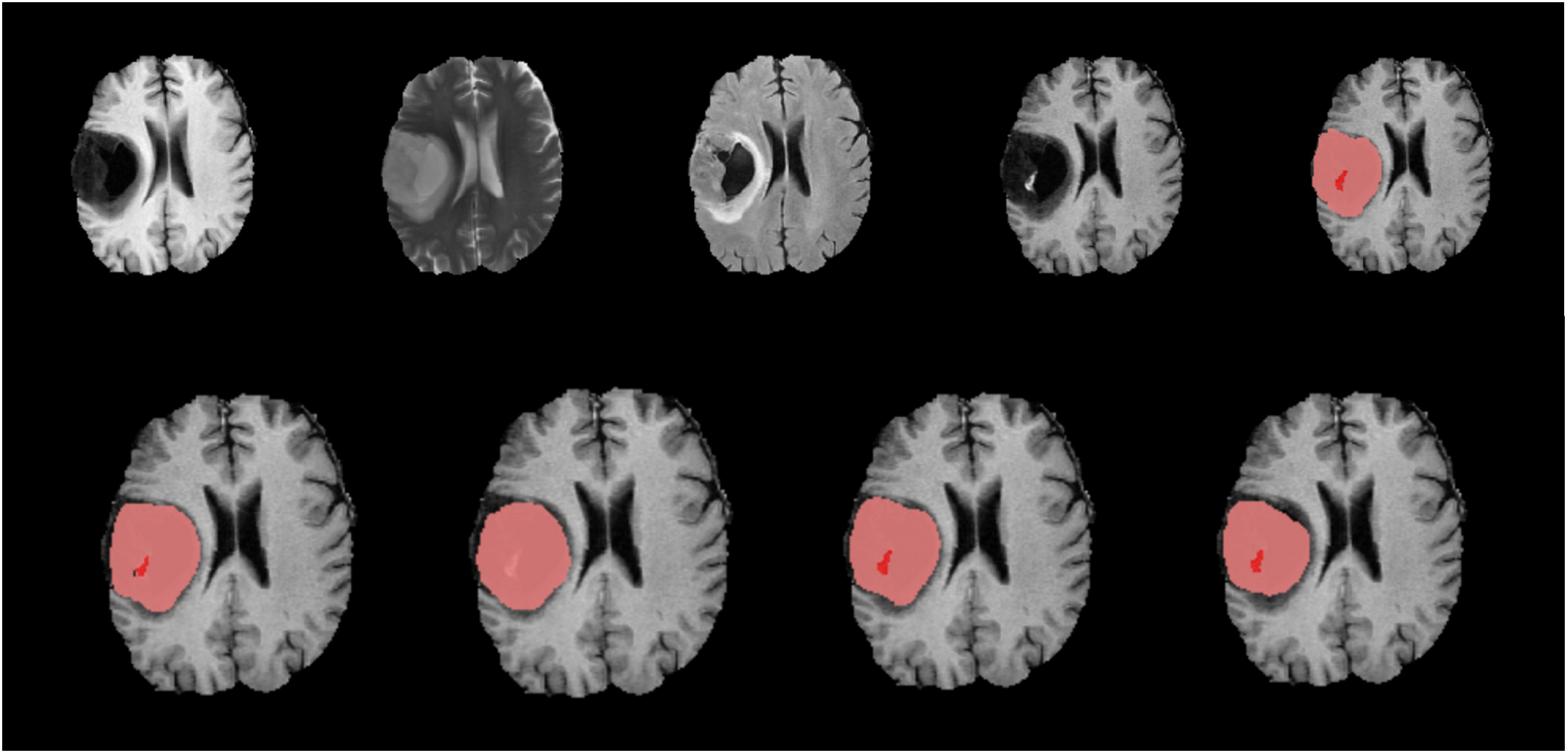
Native images of a LGG located in the right hemisphere are seen on top row, T1, T2, FLAIR, T1C and ground truth segmentation from left to right. Bottom row shows predicted segmentation masks from left to right T1C-only, FLAIR-only, doublet (T1C + FLAIR) and quadruplet (T1 + T2 + FLAIR + T1C). Red focus represents the ET where pink + red together are TC. Dice scores are; ET = 0.403/TC = 0.901 for ‘T1C-only’, ET = 0/TC = 0.826 for ‘FLAIR-only’, ET = 0.605/TC = 0.864 for doublet and ET = 0.618/TC = 0.802 for quadruplet. T1C + FLAIR and ‘T1C-only’ masks seem to capture the TC and ET very efficiently compared to the ground truth segmentation. Inability of ‘FLAIR-only’ sequence to detect the ET is noteworthy.

**Figure 6:**
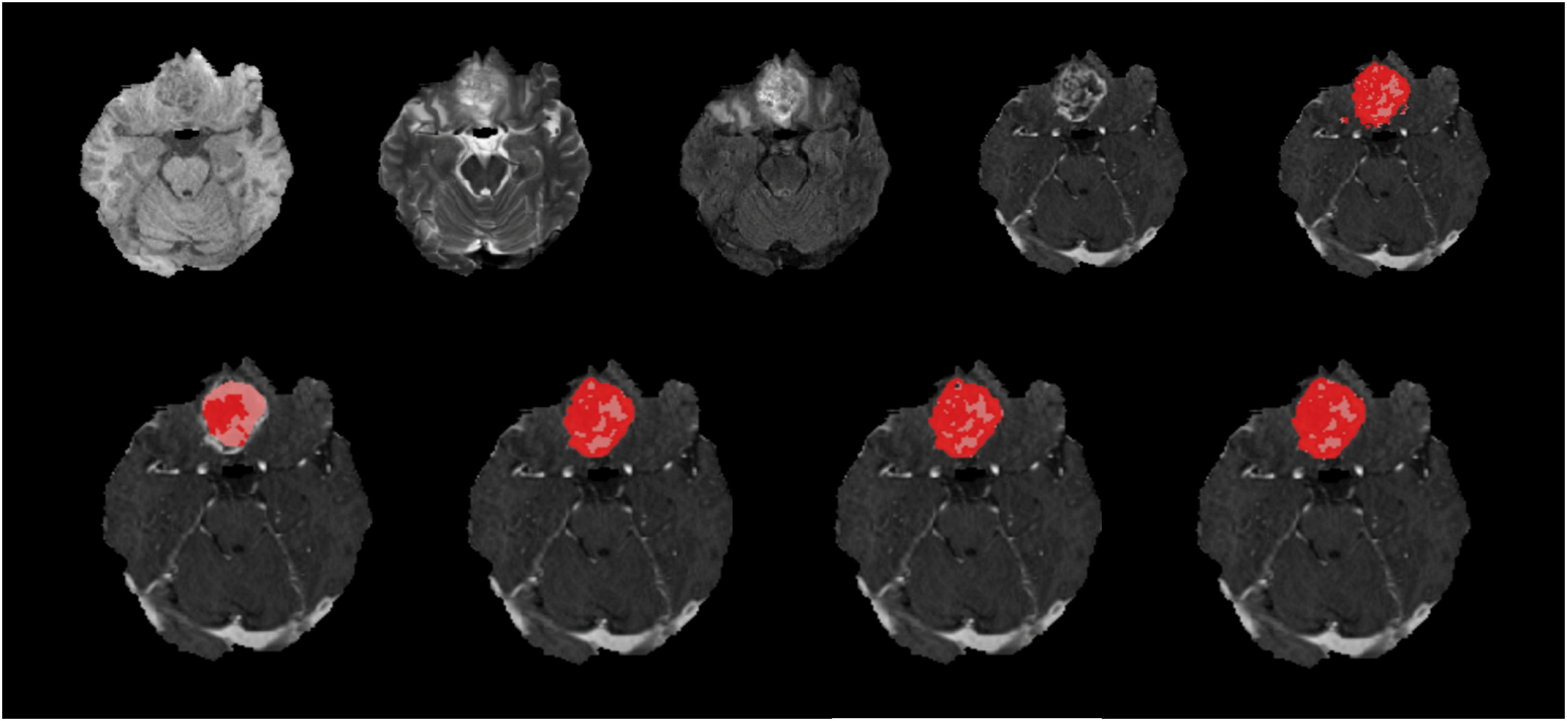
Native images of a HGG located at the right frontobasal region are seen on top row, T1, T2, FLAIR, T1C and ground truth segmentation from left to right. Bottom row shows predicted segmentation masks from left to right, FLAIR-only, T1C-only, doublet (T1C + FLAIR) and quadruplet (T1 + T2 + FLAIR + T1C). Red areas represent the ET where pink + red together are TC. Dice scores are; ET = 0.452/TC = 0.872 for ‘FLAIR-only’, ET = 0.876/TC = 0.943 for ‘T1C-only’, ET = 0.880/TC = 0.946 for doublet and ET = 0.887/TC = 0.946 for quadruplet. T1C + FLAIR mask seems to capture the TC and ET very efficiently compared to the ground truth segmentation with high Dice score. ‘FLAIR-only’ sequence missed some of the relatively less enhancing portions of the tumor.

FLAIR was the worst performing in both ET and TC segmentation. The overall poor performance when attempting to segment the ET on the training data (Dice score 0.008) contrasted with the high performances of T1C, doublet, and quadruplet models (one-way ANOVA *p* = 0.007) (Table 2). Performance on the test dataset remained poor (Dice score 0.056). We observed no correlation between the number of epochs and performance in ET segmentation using FLAIR-only; the model exhibited an inability to learn from the training data as performance did not improve with increased time (Figure 4). In TC segmentation, FLAIR consistently performed poorly compared with the three other models (Dice score 0.620; one-way ANOVA *p* = 0.01, Table 2). Examples of LGG (Figure 5) and HGG (Figure 6) are demonstrated both with native images, ground truth segmentations and predicted tumor segmentation masks.

**Table 2:** Summary of one-way ANOVA (alpha = 0.05, assuming unequal variances) for across-group comparison of performances in cross-validation for enhancing tumor (ET) and tumor core (TC) tumor segmentations.

MRI subset combinations	ET (*p*-value)	TC (*p*-value)
T1C vs. doublet vs. quadruplet	0.33	0.62
FLAIR vs. T1C vs. doublet vs. quadruplet	0.007	0.01

There was no statistically significant difference in performances across T1C, the doublet (T1C + FLAIR), and the quadruplet combinations (T1 + T2 + T1C + FLAIR), suggesting their comparability. Adding FLAIR to the comparison led to a statistically significant difference in both ET and TC segmentations, highlighting its notably different level of performance.

In a secondary set of evaluations, we examined sensitivity, specificity and 95% Hausdorff distance (Table 3). For EC, the highest sensitivity (0.828) and lowest Hausdorff distance (5.964) were observed with T1C and T1C + FLAIR, respectively, indicating superior boundary agreement and detection performance with minimal datasets. FLAIR-only subset resulted in markedly lower sensitivity (0.123) and significantly higher Hausdorff distance (170.211), suggesting poor delineation accuracy. The inclusion of all sequences yielded similar to slightly lower sensitivity (0.716) than with the doublet (0.75), but the spatial agreement (Hausdorff distance: 75.420) was better than for FLAIR. For TC, the highest sensitivity (0.754) was achieved using the quadruplet, followed closely by T1C (0.737), whereas FLAIR-only segmentation again showed poor performance (sensitivity: 0.06). Hausdorff distances in TC exhibited a narrower range (17.622–33.811) across the sequence combinations than with ET. Across all configurations, specificity remained high (≥0.958), indicating strong ability to correctly identify non-tumoral areas.

**Table 3:** Additional evaluation metrics on the test dataset across all four sequence subsets of interest in both ET (enhancing tumor) and TC (tumor core) segmentation.

Segmentation Type	Sequence subset	Sensitivity	Specificity	Hausdorff distance (95%)
Enhancing tumor	T1C	0.82889	0.99579	6.34384
Enhancing tumor	FLAIR	0.1235	0.9983	170.2105
Enhancing tumor	Doublet	0.74958	0.99653	5.96402
Enhancing tumor	Quadruplet	0.71641	0.95821	75.41999
Tumor core	T1C	0.73736	0.99797	26.84868
Tumor core	FLAIR	0.06061	1	17.62156
Tumor core	Doublet	0.6719	0.99724	33.81173
Tumor core	Quadruplet	0.75446	0.99386	22.29662

Doublet represents T1C + FLAIR, and quadruplet represents all four sequences (T1 + T2 + T1C + FLAIR). The values for sensitivity, specificity and 95% Hausdorff distance were obtained from the CBICA portal.

Similar to assessment by Dice scores, T1C and T1C + FLAIR exhibited strong overall performance in ET delineation by sensitivity, specificity, and Hausdorff distance, indicating excellent boundary agreement, identification of non-tumoral areas, and spatial accuracy. These results suggest that integrating both structural (T1C) and edema-sensitive (FLAIR) information enhances the model's ability to accurately detect and delineate ET regions. By these metrics, T1C also performed well in the delineation of TC; however, the inclusion of all sequences did lead to slight improvement. FLAIR by itself consistently underperformed in these categories except by Hausdorff distance and specificity in TC delineation, where it was positioned within range of other configurations.

We further observed that model convergence was achieved earlier than our pre-designated epoch of 60 for T1C, the doublet, and the quadruplet sequences. At approximately epoch 15, T1C and the doublet achieved high performance (Dice score >0.750) for ET, while the doublet and quadruplet achieved similar high performance for TC (Figure 4). T1C achieved high performance in ET segmentation before epoch 10, but with episodic instability at epochs 40 and 55. In TC segmentation, T1C demonstrated high performance prior to epoch 10, but convergence was not achieved until epoch 40. These results suggest that extending the number of epochs for longer training period is not expected to improve our findings.

Our study findings suggest that reducing the sequence dependency to T1C + FLAIR can provide robust segmentation performance while potentially reducing acquisition time and computational load.

## 4 Discussion

Although brain tumor segmentation by AI technology has been a key topic for the last 10 years with substantial amount of research focus, findings demonstrated in this study offer a different point of view by testing whether smaller subsets of MRI sequence data are sufficient for a DL model to achieve high-performance segmentation of subregions of glioma on 3D brain MRI images. Our study, intentionally scoped to balance methodological rigor with practical applicability, confirmed that a doublet (T1C + FLAIR) sequence combination could achieve a tumor segmentation performance comparable to—and, in some cases, even surpassing – that of the full four-sequence model.

Although highly accurate DL-based models have been tested on comprehensive MRI sequence sets, to our knowledge, no study has demonstrated the ability to attenuate such data-intensive requirements with the use of a doublet MR sequence, specifically T1C + FLAIR, for glioma segmentation (15, 26, 27). Moreover, no previous study has addressed the challenge of minimizing the data without compromising performance to achieve high performance brain tumor segmentation from a clinical or neuroradiological perspective**.** Dehghani et al. conducted a similar study comparing different sequence combinations using the BraTS 2020 challenge dataset and stated that FLAIR sequence is the best choice for a single sequence while joint segmentation on the entire four MR sequences would yield higher accuracy (13). We defined the whole tumor area including enhancing and non-enhancing parts as ‘tumor core' unlike the given study assessing only the enhancing parts with T1C images, which we believe is the cause of the discrepant results. Additionally, their study differed from our study in a few notable ways, including execution of a simpler task that did not include tumor subregion segmentation (TC and ET). Also, in contrast to our study, it performed single training on each model to be applied on a held-out test set, rather than seek model optimization through cross-validation, then rigorously test the model on an independent test dataset. Furthermore, our study used the MICCAI-hosted evaluation portal for assessment of accuracy on 66 cases (18%) of the 2018 BraTS validation dataset to minimize bias.

The ability to use smaller sequence subsets with adequate segmentation performance can be leveraged in real-world clinical and research settings by reducing the data requirement, enhancing generalizability and promoting dissemination of DL algorithms in real-world settings, where resources may be limited and full MRI scans are unavailable. By overcoming a well-known barrier to DL algorithm use - its dependence on data-rich training sets - the ability to leverage smaller datasets reduces the training time and computational burden. It also lowers the requirement for reduced sequences used for training.

T1C-only was expected to demonstrate lower performance in LGGs due to lack of contrast enhancement. Indeed, Dice scores were lower in delineating ET-specific regions in LGGs compared to HGGs. However, the global performances of ET and TC segmentations were high regardless of tumor enhancement. Even non-enhancing tumor parts were successfully depicted by T1C alone. This performance may be attributable to high training data quality owing to meticulous ground truth annotation by experts and data diversity inclusive of a spectrum of representative cases (16). Relatively lower Dice scores in LGGs may be due to the challenge of the model in differentiating peritumoral edema and non-enhancing tumor from each other while still adequate to show accurate performance for segmentation of the whole tumor (13). T1C + FLAIR overcame the lower capacity of T1C in the detection of LGGs making it the best sequence combination for the accurate segmentation of both LGGs and HGGs.

FLAIR-only is ineffective for ET and TC segmentation and inferior to other sequences as the signals captured do not accurately represent the subregions of interest, likely due to the expected decreased contrast-to-noise ratio (24). The BraTS dataset consisted of 74% (*n* = 210) HGGs and 26% (*n* = 75) LGGs where the former were historically defined by contrast enhancement, and the latter typically poorly visualized with contrast, while better captured by FLAIR. Nevertheless, LGGs did not emerge as failure cases in these experiments, and there were no cases where FLAIR consistently outperformed T1C. In TC segmentation, however, FLAIR-only was at the higher end within a range of other configurations by Hausdorff distance and specificity. It is still possible that the superior performance of T1C containing subsets may not persist with a higher composition of LGGs, a hypothesis that warrants future investigation using a dataset with a lower proportion of HGGs. However, we also do not have verification that high and low-grade labels provided by BraTS were defined based on the degree to which tumors do or do not enhance with contrast.

Performance of the quadruplet model in ET segmentation was erratic, performing worse than both T1C and doublet until epoch 60. It is possible that the quadruplet model did not converge at 60 epochs and running to higher epochs would have yielded better results, although it would incur greater computational burden compared to other models.

A possible limitation of this study is related to the anatomical distribution of the tumor cases included. All the cases analyzed were sourced from the BraTS Challenge datasets 2018 and 2021, which exclusively feature supratentorial gliomas (4, 21). Consequently, the segmentation performance of the proposed approach has not been assessed for tumors located in more complex regions such as the posterior fossa or brainstem. Future studies including infratentorial tumor cases are warranted to assess the generalizability and robustness of the method across a wider anatomical spectrum.

## 5 Conclusion

Limited brain MRI sequences, such as T1C + FLAIR can achieve consistently high-performance tumor segmentation, comparable to—and more efficiently than—comprehensive quadruplet sequences. Our findings overcome both the barriers of data-intensive requirements of DL algorithms and data availability in community or resource-constrained real-world clinical and research settings, where the acquisition of multiple-sequence MRI scans for each patient can be cost-prohibitive and time-consuming. With our key finding that T1C + FLAIR doublet can achieve comparable performance to larger number of sequences, we provide a practicable means by which automated tumor segmentation can be disseminated and become a globally accessible tool, including in environments facing reduced resource availability. Ultimately, demonstration of feasibility and practicability, as we have sought in our study, can critically impact the adoption of artificial intelligence to meet health care needs.

## Data Availability

Publicly available datasets were analyzed in this study. This data can be found here: CBICA Image Processing Portal, https://www.med.upenn.edu/cbica/brats2021/#Data2. Access to the dataset should be requested directly from the organizer.
